# Effects of Physiotherapy vs. Acupuncture in Quality of Life, Pain, Stiffness, Difficulty to Work and Depression of Women with Fibromyalgia: A Randomized Controlled Trial

**DOI:** 10.3390/jcm10173765

**Published:** 2021-08-24

**Authors:** Elisa María Garrido-Ardila, María Victoria González-López-Arza, María Jiménez-Palomares, Agustín García-Nogales, Juan Rodríguez-Mansilla

**Affiliations:** 1 ADOLOR Research Group, Department of Medical-Surgical Therapy, Medicine Faculty, Extremadura University, 06006 Badajoz, Spain; egarridoa@unex.es (E.M.G.-A.); mariajp@unex.es (M.J.-P.); jrodman@unex.es (J.R.-M.); 2 Mathematics Department, Medicine Faculty, Extremadura University, 06006 Badajoz, Spain; nogales@unex.es

**Keywords:** fibromyalgia, core stability, acupuncture, quality of life, pain, stiffness, work impairment, depression

## Abstract

Background: Fibromyalgia is a chronic clinical condition characterized by pain and other associated symptoms that have a negative impact on the quality of life of the affected person. The objective of this study was to assess the effectiveness of a core stability training physiotherapy program compared to an acupuncture treatment on quality of life, pain, joint stiffness, difficulty to work and depression of women with fibromyalgia. Methods: This was a single-blind, randomized clinical controlled trial. Women with fibromyalgia were randomized to a core stability physiotherapy program group (*n* = 45), an acupuncture treatment group (*n* = 45) and a control group (*n* = 45) for 13 weeks. Measurements were taken at baseline (week 0), post-intervention (week 6) and at follow-up (week 13). The primary outcome measure was quality of life (Spanish Fibromyalgia Impact Questionnaire). The secondary outcome measures were pain, joint stiffness, difficulty to work and depression (Visual Analogue Scale). Results: In total, 103 participants completed the study. The results, from a descriptive perspective, showed improvements in all the outcome measures in both intervention groups (physiotherapy and acupuncture) at weeks 6 and 13 in relation to week 0 and in comparison to the control group. Only the difficulty to work measure in the acupuncture group showed a slight decrease at week 13. In particular, mean (±SD) Spanish Fibromyalgia Impact Questionnaire score at 6 weeks was 62.89 ± 16.91 for the physiotherapy group, 62.5 ± 18.09 for the acupuncture group and 67.45 ± 17.07 for the control group. However, these improvements were not statistically significant. Conclusion: Core stability-based physiotherapy and acupuncture showed non-significant improvements in quality of life, pain, joint stiffness, difficulty to work and depression in women with fibromyalgia.

## 1. Introduction

The main clinical manifestation of fibromyalgia is the presence of diffuse and widespread pain in combination with multiple tender points [1]. Although pain is the most frequent symptom [2], it generally appears to be associated with other symptoms. These include stiffness; fatigue [2,3]; sleep disturbances [2]; balance [2,4] and functionality impairments [5]; cognitive disorders [5,6], such as difficulty to concentrate, confusion, memory lapses or difficulty in verbal fluency; and depression [2,3], among others. As a result, patients also report difficulties in performing their activities of daily living [7,8] and impaired ability to work [9,10].

Fibromyalgia has significant psychosocial and economic repercussions, being one of the rheumatic diseases with the greatest impacts on quality of life [11,12] and health status [13]. Both concepts are directly interrelated and often refer to health-related quality of life [8]. Consequently, this condition generates a high consumption of care resources and high personal, social and employment costs in those patients with fibromyalgia [14].

Exercise programs have been consistently recommended for the management of fibromyalgia [15,16]. Likewise, exercise is one of the treatment approaches used by physiotherapists in their clinical practice [17]. As defined by the World Confederation of Physical Therapists [17], physiotherapy comprises ‘services provided by physical therapists to individuals and populations to develop, maintain and restore maximum movement and functional ability throughout the lifespan. The service is provided in circumstances where movement and function are threatened by ageing, injury, pain, diseases, disorders, conditions and/or environmental factors with the understanding that functional movement is central to what it means to be healthy’. In particular, exercise programs based on the training of core stability are among the newest therapeutic exercise modalities used in physiotherapy for the treatment of various pathologies. This type of training has recently become popular and is beginning to have a great impact on rehabilitation [18]. The main objective of this type of exercise is the strengthening the core muscles [19] in order to restore muscle balance [20]. Core stability training focuses on the rehabilitation of movement dysfunction and is based on four well-defined principles: core muscle activation, breathing, correct alignment and control and fluidity of movement [21].

The most recent revision of the European League Against Rheumatism (EULAR) recommendations for managing fibromyalgia [22] indicated that exercise is the only ‘strong for’ therapy-based recommendation in the guidelines. Furthermore, recent systematic reviews and meta-analyses have shown that exercise improves pain [23,24,25], physical function [23,24], stiffness [23], depression [25], fatigue [23,26], global well-being [25] and health-related quality of life [23,25]. In particular, core stability training has been shown to reduce pain and improve quality of life in women with different conditions [27,28]. In addition, other authors have found positive effects of this type of exercise on physical function and flexibility [29], mood states [30] and depression symptoms [31]. There are few core stability training interventions available in the literature that have been conducted on women with fibromyalgia ad that analyzed our variables of interest [32,33]. These studies have shown positive changes in relation to pain, anxiety and quality of life after completing the treatment [32,33].

Another non-pharmacological treatment approach recommended for the management of fibromyalgia is acupuncture [34]. The World Health Organization defines acupuncture as the insertion of needles into humans or animals for therapeutic purposes [35]. This traditional Chinese medical technique consists of placing very fine needles on specific points on the body along the body’s energy channels, also called meridians, for a maximum of 30 min [36,37]. Once placed, the needles can be manipulated manually with electro-stimulation (electro-acupuncture) or heat with mugwort cigars or cones (moxibustion) in order to balance the body’s energy or qi [36]. Different reviews support the use of this therapy based on its effects, improving pain [38,39,40,41], fatigue, stiffness [38] and quality of life [41] in comparison to conventional treatment, sham acupuncture, no treatment or drug therapy.

The evidence available suggests the potential that core stability training and acupuncture have for the treatment of women with fibromyalgia. However, no studies that compare both treatment approaches and analyze their effects on pain, stiffness, difficulty to work, depression and quality of life have been found in the literature.

The objective of the present study was to assess the effectiveness of a core stability-based physiotherapy program vs. an acupuncture treatment regime in improving pain, stiffness, difficulty to work, depression and quality of life, comparing both treatment approaches and a control group.

## 2. Materials and Methods

### 2.1. Design

A single-blind, randomized controlled clinical trial was conducted in the University of Extremadura and the Fibromyalgia Associations from Badajoz and Olivenza in Extremadura (Spain) in an outpatient setting. The CONSORT Statement was used to conduct and report the trial. The trial was registered at ClinicalTrials.gov (study identifier: NCT03638518). Ethical approval was received from the Bioethical Commission of the University of Extremadura in Spain (registration number: 79/2013). Written informed consent was signed by all the participants.

### 2.2. Participants

The population of interest was women diagnosed with fibromyalgia from the mentioned Fibromyalgia Associations. The inclusion criteria were women between 18 and 71 years old, diagnosed with fibromyalgia by a specialized physician. Patients were excluded if they had any medical contraindication for acupuncture and/or physiotherapy, phobia of needles, adverse reactions to medication that could influence the effect of the treatment, associated pathologies such as alcoholism or severe visual deficit, received acupuncture or core stability-based physiotherapy in the two months prior to the intervention and if they had performed any core stability exercise modalities such as Pilates or yoga.

**Figure 1:**
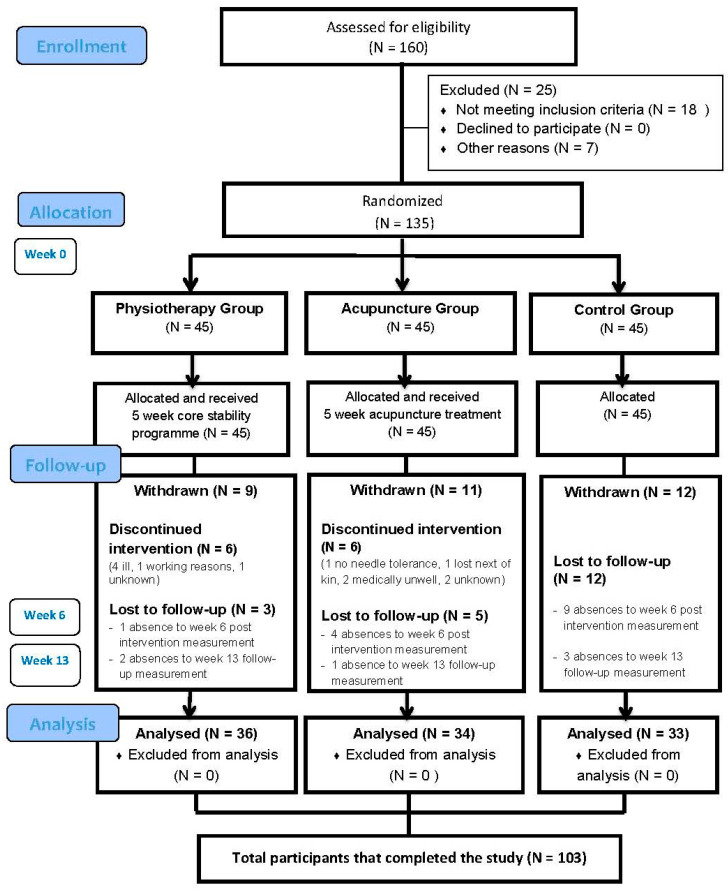
CONSORT flow diagram of study participation.

The participants who met the selection criteria (*n* = 135) were randomly allocated to a physiotherapy experimental group, an acupuncture experimental group or a control group (Figure 1). The randomization was carried out by an independent researcher who was unrelated to any aspect of the trial. No-one directly involved in the project had access to the randomization process or the list. In brief, 135 sealed envelopes containing the names of the three study groups were introduced in an opaque bag. The person responsible for the randomization kept the bag closed and only opened it when each participant took an envelope out. The allocation of the participants was concealed until assignment. After the baseline assessment, this person informed the participants of the group to which they were allocated.

### 2.3. Data Collected and Outcome Measures

All participants attended three measurement sessions: one on entry into the study and prior to randomization (baseline—week 0), one after 5 weeks of treatment (post-intervention—week 6) and one after a 5-week follow-up period (follow-up—week 13). The University of Extremadura laboratory was the location where all measurement sessions took place. The assessor was a physiotherapist blinded to group allocation at all times. He was independent to the study and was not aware of the treatments applied or the objective of the therapy. Neither the participants nor their therapists were blind to the group assignment. Due to the nature of the treatment, they could clearly see which group the participant was allocated to.

A protocol was established to collect the data. The sociodemographic data that were collected included age, residence, education, working status/employment situation and concomitant treatment (medication).

The primary outcome measure was quality of life, assessed with the Spanish Fibromyalgia Questionnaire (S-FIQ) [42]. This questionnaire is an adaptation of the Fibromyalgia Impact Questionnaire developed by Burckhardt et al. [43] in 1991. The S-FIQ has a reliability coefficient of 0.81. The maximum score is 100, and the higher the result obtained is, the higher the impact of the condition on the person is.

The secondary outcome measures were pain, stiffness, difficulty to work and depression, which were measured with the Visual Analogue Scale (VAS) [44]. This scale is a simple, robust, sensitive and reproducible instrument that was developed by Huskisson [45] in 1974. According to the medical evidence, it is the most frequently used measurement method for pain assessment. Patients were asked to rate their perception of pain intensity, stiffness level, difficulty to work and depression state using a 10-point scale, where 0 indicated ‘no symptoms’ and 10 indicated ‘extreme and unbearable symptoms’ [44].

Data related to any possible adverse events for both experimental groups were also collected by the physiotherapist and the physician who conducted the treatment. In addition, reasons for missing any outcome measurement or treatment sessions were also recorded.

### 2.4. Interventions

The patients allocated to the physiotherapy experimental group followed a core stability training program, those allocated to the acupuncture experimental group received acupuncture treatment and those allocated to the control group did not receive any intervention. Details of the intervention following the Template for Intervention Description and Replication (TIDieR) guidelines [46] are provided in Supplementary Table S1. The study was conducted over 13 weeks, comprising 5 weeks of treatment, 5 weeks of follow-up and 3 weeks of measurements. During the study, all participants continued with their routine medical treatment, complying with the beneficence and non-maleficence principles of bioethics.

### 2.5. Statistical Analysis

The obtained data were analyzed using SPSS version 19.0 (Statistical Package for the Social Sciences). A descriptive analysis of the characteristics and the baseline measurements of the participants was performed. Their homogeneity was verified with an analysis of variance (ANOVA). A mixed-model analysis was applied to compare the obtained values of the quantitative variables of the three study groups (physiotherapy, acupuncture and control) at the three measurement times (baseline, post-intervention and follow-up). Furthermore, differences between groups at baseline, week 6 and week 13 were calculated with a one-way ANOVA (F-test). Whether variances could be considered as equal or not equal was taken into account in the analysis.

A value of *p* < 0.05 was considered statistically significant for all tests. As a comparison was made between two groups, an absolute measure of the effect size (mean difference) was determined to complement the *p*-value. This method was considered to be the most appropriate to analyze the effect of the treatments applied.

The study sample size was not formally calculated due to the fact that the study relied on the availability of the members of the Fibromyalgia Associations to participate. Based on previous studies of our research group and the statistical guidelines [47], a minimum of 25 or 30 participants recruited per group was anticipated and would be enough to justify the use of the statistical methods actually employed.

## 3. Results

A total of 160 women with fibromyalgia were assessed for eligibility for the trial over a 12-month period. As can be observed in Figure 1 (CONSORT flow diagram), 135 participants were recruited and randomized into the three study groups and 103 participants completed the study.

The groups appeared homogeneous in terms of baseline characteristics and baseline scores on the outcome measures (Table 1). There were no statistically significant between-group differences.

Table 2 summarizes the results of the outcome measures (in the format mean ± SD) for the three groups at weeks 0 (baseline), 6 (post-intervention) and 13 (follow-up). In order to analyze between-group differences at weeks 0, 6 and 13, a one-way ANOVA was carried out for each measurement time (Table 3). All the outcome measures showed improvements in both intervention groups (physiotherapy and acupuncture) at weeks 6 and 13 compared to week 0 and in comparison to the control group. Only a slight decrease was observed in the acupuncture group regarding the variable ‘difficulty to work’ when comparing weeks 0 and 13. However, no statistically significant results were found for the primary outcome measure of the study or for any of the secondary outcome measures. Therefore, none of these variables could be used to analyze between-group differences at baseline, after the treatment or at follow-up.

Moreover, Table 4 presents the results of the mixed model analysis carried out in order to assess the changes experienced by each group during the study period at weeks 0, 6 and 13. None of the three groups considered showed statistically significant changes throughout the study period for any of the variables of interest.

In relation to adverse events during the intervention in the acupuncture group, only one event was reported and registered. One woman experienced knee pain exacerbation due to her arthritis and had to rest during the last three sessions of the physiotherapy treatment.

## 4. Discussion

The results of our study show that the core stability-based physiotherapy program and the acupuncture treatment improved the scores obtained for quality of life, pain, joint stiffness, perceived difficulty to work and depression in women with fibromyalgia after the treatments were completed in comparison with the control group. However, the changes were not statistically significant. Considering the chronic nature of fibromyalgia and its clinical evolution, which implies fluctuation of symptoms and significant variations during the course of the disease [48], we consider our results to be relevant, as the treatments applied can help to maintain the quality of life of the affected person.

The quality of life and impact of fibromyalgia index in our study was 67.87 on average across all study participants at baseline. According to the criteria established by Bennet et al. [49], this indicates a severe impact of the disease. In addition, this value is close to 70, a figure that Monterde et al. [43] established as indicative of severe cases of fibromyalgia. Our results in relation to the initial S-FIQ scores of the participants are slightly lower than those in the EPIFFAC study [50], in which the mean S-FIQ score in a representative sample in Spain was 75.5.

We observed, from a descriptive point of view, a decrease of 7.11 and 6.47 points in the S-FIQ scores in the acupuncture and physiotherapy groups, respectively, after application of the treatments, which indicates a slight, non-significant improvement in comparison to the control group. These positive changes were maintained in the follow-up measurement after the rest period. Several systematic reviews [23,24,25,26] analyzed the efficacy of physical exercise programs and confirmed that physical exercise and physiotherapy improve the quality of life of patients with fibromyalgia [24,25]. In relation to core stability exercises, we found that there is a dearth of studies that assess the efficacy of core stability exercise training programs in the management of fibromyalgia symptoms. Altan et al. [33] and Ekici et al. [32] assessed the effects of these types of exercises in comparison to a home exercise relaxation/stretching program and to connective tissue massage, respectively. Both studies [32,33] conducted a Pilates core stability exercise program which consisted of 1-hour sessions, performed three times a week. Altan et al. [33] performed the program for 12 weeks, while Ekici et al. [32] carried out the exercises for 4 weeks. Their results coincide with our findings as the S-FIQ scores decreased in the core stability group after the intervention [32,33] and after the follow-up period [33]. However, their improvements indicated statistically significant improvements, while ours suggest that core stability helped to maintain the quality of life.

In comparison to the results obtained by Altan et al. [33], we consider that the differences regarding the significance of the results could be due to the difference in the duration and the lower frequency of sessions. In our study, the interventions were carried out for 5 weeks in order to allow the experimental groups to be treated equally. This is the length of time needed to complete an acupuncture course of treatment and also respects the minimum recommended for fibromyalgia exercise programs [51]. Nevertheless, our sample size was considerably larger than the samples in the mentioned studies.

Our exercise program was based on core stability training. This type of exercise aims to improve proximal stability to allow better upper and lower limb movement [52]. Proximal stability is essential for correct body function. If the stabilization system does not function and there is no balance between stabilizing and mobilizing muscles, muscle imbalance and movement dysfunction will occur. This will lead to mechanical stress on bone structures and the neuromuscular system and will also have a negative impact on pain, joint stiffness and general well-being, among others [53]. Therefore, we consider that our core stability program could have contributed to controlling the negative effects of muscle imbalance and, hence, showing a slight improvement in the S-FIQ scores of the participants in the physiotherapy group, although the statistical analysis indicated that it is not significant.

Two meta-analyses [40,41] published in 2019 studied the effectiveness of acupuncture in improving the quality of life among other symptoms of patients with fibromyalgia. Zhang et al. [41] analyzed studies that compared acupuncture therapy to sham acupuncture or conventional medication, while Jiwon et al. [40] analyzed the effects of verum acupuncture vs. sham acupuncture. Both authors [40,41] concluded that real acupuncture was significantly better than sham acupuncture for improving quality of life after treatment. In addition, the results obtained by Zhang et al. [41] showed that this evidence was moderate and that, in the long term, real acupuncture had a superior effect on improving the quality of life compared with sham acupuncture. Regarding the effects of the acupuncture treatment on the quality of life measured with the S-FIQ, the decrease in the scores obtained in our study does not coincide with the improvements observed in the most recent reviews available in the literature.

Furthermore, authors such as Schweiger et al. [54] have also evidenced the effectiveness of acupuncture treatment in women with fibromyalgia. They conducted a randomized clinical trial with 60 women and compared the effects of acupuncture vs. treatment with a nutritional supplement combination. The acupuncture treatment was individualized and was performed at a frequency of twice a week for 3 months. The results showed that the acupuncture treatment significantly improved the quality of life of the participants after 1 month of treatment and after completing the treatment. Additionally, the positive changes were maintained after a follow-up period of 3 months. Again, our results do not coincide with those of Schweiger et al. [54]. This could be explained by the differences in the length of the treatment applied. We conducted one course of acupuncture treatment (two sessions per week for a total of 10 sessions), while Schweiger et al. [54] conducted two courses of acupuncture after an interval of at least 1 month.

It is interesting to highlight that the three points that were used in our study (GV20, ST36 and BL60) are also among the most recurrent acupuncture points that Schweiger et al. [54] applied in their treatment. GV20 is indicated to increase energy (qi) levels. ST36 harmonizes the energy and has toning and body-strengthening effects. Therefore, it is indicated for the treatment of disorders that present fatigue and weakness. BL60 facilitates the flow of the traditional Chinese medicine channels and it regulates and tones the musculoskeletal system [36,37]. All the effects attributed to these acupuncture points can contribute to improving the well-being of patients with fibromyalgia and, consequently, their quality of life. Furthermore, it has been observed that plasma and brain tissue levels of endomorphin-1, beta-endorphins, encephalin and serotonin increase with the application of acupuncture, which causes analgesia, sedation, recovery of motor functions and an immunomodulatory effect. It is also thought that at the sensory level, it stimulates mechanoreceptors and inhibitory neurons, which causes a decrease in pain transmission. In turn, at the cognitive level, it would change the focus of attention and induce a feeling of self-efficacy in the person [55]. Therefore, we consider that the benefits obtained by the participants of the acupuncture group could be explained by the effects of the acupuncture points that were used in our study. In addition, both in the study by Schweiger et al. [54] and in our study, achievement of the deqi sensation was sought after insertion of the needle. This sensation has been clinically related to the effectiveness of acupuncture [56,57] and could also have contributed to the decrease in the S-FIQ scores in the acupuncture group.

We believe that the results of the S-FIQ observed in the two experimental groups are related to those obtained in the secondary outcome measures. In this respect, different authors have evidenced the relation between fibromyalgia symptoms and quality of life. Mease [11] highlighted that pain, disability and other symptoms of fibromyalgia significantly reduce quality of life. Additionally, Arnold et al. [8] conducted a study where fibromyalgia patients identified the symptom domains that had the greatest impact on their quality of life. They included pain, sleep disturbance, fatigue, depression, anxiety and cognitive impairment.

Although pain is the most frequent symptom in fibromyalgia [2], stiffness is reported in 76–84% of patients [2,3]. The mean values of pain and stiffness at baseline were 7.16 and 6.98, respectively. These figures of the physiotherapy and acupuncture groups decreased slightly after the treatments from a descriptive point of view in comparison with the control group, which did not experience changes throughout the study. However, the changes observed in the experimental groups were not significant. In relation to the physiotherapy group, our results agree with those of Ekici et al. [32], Altan et al. [33] and Martínez-Rodríguez et al. [58] in that the scores of pain and stiffness decreased after treatment. However, they do not agree in terms of the significance of the changes. As in our study, the two latter groups also found that the pain relief was superior in the group that completed the core stability program in comparison to the control group. Scientific evidence supports that exercise leads to the production of endorphins that have a pain-inhibiting effect [59,60]. This would explain the slight analgesic effect obtained after the implementation of the core stability exercise program. We also consider that the muscle balancing and strengthening effect could have contributed to better alignment of the joints, therefore decreasing mechanical stress on bone structures and the neuromuscular system and slightly decreasing the stiffness and pain scores [53]. However, pain in fibromyalgia is considered to be multifactorial and to present a persistent alteration in pain regulation systems as well as a decrease in the modulation of response inhibition activity and pain control [61]. This could explain why the treatments did not achieve better results.

At 6 weeks, the participants of our study that received acupuncture treatment showed non-significantly lower levels of pain and stiffness which were maintained after the follow-up period. Our results are not consistent with those obtained in the reviews conducted by Jiwon et al. [40], Zhang et al. [41] and Deare et al. [38], who concluded that real acupuncture is an effective treatment for pain relief in fibromyalgia with low- to moderate-quality evidence. Deare et al. [38] also concluded that acupuncture significantly improves pain and stiffness compared to conventional treatment or no treatment. It should be noted that our sample size was comparable to the larger ones in their review (between 4 and 36 participants per study group). In addition, as in our study, no patients reported severe adverse effects of acupuncture and the authors considered the technique safe.

Recent randomized controlled trials have also evidenced the benefits of acupuncture for pain relief of fibromyalgia patients [62,63,64]. Vas et al. [62] and Ugurlu et al. [63] compared the effects of real acupuncture and sham acupuncture, while Mist et al. [64] compared acupuncture with group education. Their results showed statistically significant differences when measuring pain levels after treatment. Our results therefore agree in relation to the positive changes in pain scores but not in terms of the significance of the improvements. Based on the statements of Cabyoglu et al. [55], we believe that the analgesia achieved with acupuncture treatment could be due to the release of endorphins that this treatment produces. We also believe that puncture of the GV20 point could influence pain levels. Its shen-pacifying and brain-toning effect [36] can have a positive effect on the psychological components of pain in fibromyalgia.

When comparing acupuncture with drug treatment, such as amitriptyline or fluoxetine, it has been shown that acupuncture is more effective in relieving pain in people with fibromyalgia and that the combination of medication, exercise and acupuncture may also be beneficial [65]. In our study, acupuncture was compared with core stability exercises, but it would be interesting to see in future studies whether both therapies are more effective when used in combination.

The initial mean score obtained for the participants’ difficulty to work was 6.7 as measured with the VAS. This is consistent with the results of Collado et al. [50], Clark et al. [66] and Sallinen et al. [67], as they showed how women with fibromyalgia face limitations when carrying out their work. In relation to this outcome measure, the experimental groups showed a slight non-significant improvement that was not sustained over time. In the literature search, we found one study [32] that analyzed the effects of a core stability program on the difficulty of working in people with fibromyalgia. Ekici et al. [32] found significant improvements with both experimental treatments (core stability training and massage). However, they did not compare the results with a control group. Although this outcome measure is an item of the S-FIQ, which is used in a large number of clinical trials, a limited number of studies that analyzed the effectiveness of core stability exercises or acupuncture on the difficulty of working experienced by women with fibromyalgia were found in the scientific literature. This has made it difficult to contrast the data.

Scientific evidence indicates that psychological disorders such as depression are present in people with fibromyalgia with a prevalence of 40% [2,3]. The mean baseline value for the level of depression of the study participants as measured by the VAS was 7.12. After completing the treatments, both experimental groups showed a slight and non-significant improvement in depression level compared to the control group. The participants who completed the acupuncture treatment maintained the improvements after the rest period, even improving their scores slightly. Those who completed the core stability program worsened slightly but did not return to their initial levels. The controls had a very similar level of depression on all three measures throughout the study.

In relation to the studies available in the literature that analyzed the effectiveness of core stability exercises on levels of depression in patients with fibromyalgia, Ekici et al. [32] found significant improvements in both experimental groups, where the core stability group was superior to the massage group. Additionally, a recent meta-analysis that analyzed the effects of Pilates exercise on mental health outcomes in other types of study populations showed significant and large reductions in depressive and anxiety symptoms [31]. It is well known that exercise has beneficial effects on fibromyalgia symptoms, and authors such as Sosa-Reina et al. [25] have found strong evidence that it reduces depression among other symptoms. Different authors have suggested that the increase in serotonin levels caused by core stability training could explain the improvement of depressive symptoms in women with different conditions [31]. In addition, the breathing control performed during core stability exercises can reduce stress via activation of the parasympathetic system, which can have a positive effect on reducing depression symptoms [68].

Authors such as Jang et al. [69] and Bastos et al. [70] have shown that after completing 4 [69] or 8 weeks [70] of standardized acupuncture treatment, the depression levels of fibromyalgia patients decreased significantly. We think that the changes obtained after the application of acupuncture treatment may have been due to the antidepressant effect of point E36 [36,71]. Furthermore, although the mechanism of acupuncture seems to be unclear, there is scientific evidence that suggests this therapy approach improves serotonin levels and therefore decreases depression symptoms [72].

The changes observed in the levels of depression in both treatment groups may also be associated with the changes experienced in the other symptoms analyzed. In addition, it may also be due to the fact of attending therapy and meeting other women with the same pathology, resulting in an environment that facilitated communication and exchange of experiences about the difficulties and problems they have to face, with the consequent influence this has on emotional well-being [73].

### 4.1. Study Limitations

There are some limitations to this study. The treatment period could be considered a limitation and could explain the non-significance of the results. However, the interventions were carried out over 5 weeks in order to allow the groups to be treated equally and to ensure the methodological quality of the study. We consider that 5 weeks could have been insufficient to show significant changes in the physiotherapy group as the patients usually take 3 to 4 sessions to become familiarized with the principles of core stability exercises. This limitation was minimized with the guidance and close supervision of the physiotherapist during the sessions. Additionally, based on previous studies conducted with the same population in our region [74,75], if the period of intervention was longer, there was a higher risk of absences and decrease in adherence to the treatment.

We believe that another limitation could be the application of a standardized acupuncture treatment. According to the traditional Chinese medicine treatment principles, an individualized treatment would be more appropriate for better results [39]. However, in our study, due to the methodological quality requirements of clinical trials [76], specific points were selected and applied to all patients.

In future research, we would recommend longer treatment and follow-up periods and the use of a monitoring method. This could increase the adherence to the treatment and reduce the rate of drop-outs, which would probably reflect in the achievement of better results.

### 4.2. Clinical Implications

The results of the present study can have positive implications in the clinical practice of professionals working in the rehabilitation field. Our data show that core stability-based physiotherapy and acupuncture treatments improved the scores of quality of life, pain, stiffness, difficulty to work and depression in women with fibromyalgia from a descriptive point of view. The changes were not significant, which suggests that these techniques can maintain the mentioned symptoms and control the progression of the condition. They are two low-cost techniques that are increasingly used by health professionals and can be performed safely if they are applied by a trained professional. Core stability training can be performed by patients in groups under supervision, and once learned, it can be performed individually at home, which encourages self-management. Acupuncture is a technique that is applied twice a week and avoids the difficulty of attending a daily treatment implied in other therapies. In addition, the improvement in scores from acupuncture was maintained after 5 weeks of follow-up. This means that patients could have resting periods without losing the benefits of the treatment. Furthermore, both techniques can be combined with other therapies recommended for fibromyalgia, such as pharmacological treatment, self-management techniques, education, other types of exercise or cognitive behavioral therapy [15,77]. Therefore, the use of these treatment approaches for the management of fibromyalgia could control symptoms that have an important negative impact on the quality of life of the affected person and involves high sanitary expenditure [77].

## 5. Conclusions

Core stability-based physiotherapy and acupuncture treatments showed non-significant improvements in quality of life, pain, joint stiffness, difficulty to work and depression in women with fibromyalgia.

## Figures and Tables

**Table 1:** Baseline demographic and clinical characteristics of the participants.

Outcome	Group	*N*	Mean	Standard Deviation	95% Confidence Interval for the Mean		Sig.
Measure					Lower	Upper	
					Limit	Limit	
Age (years)	Control	33	54.39	8.20	51.49	57.30	
	Physiotherapy	36	56.06	8.37	53.22	58.89	
	Acupuncture	34	56.15	7.90	53.39	58.90	
	Total	103	55.55	8.12	53.97	57.14	0.61
Years of diagnosis	Control	33	8.30	4.54	6.69	9.91	
	Physiotherapy	36	8.03	6.30	5.90	10.16	
	Acupuncture	34	8.59	5.18	6.78	10.39	
	Total	103	8.30	5.37	7.25	9.35	0.91
S-FIQ (0–100)	Control	33	64.42	15.03	59.09	69.75	
	Physiotherapy	36	70	17.46	64.09	75.91	
	Acupuncture	34	68.97	16.98	63.05	74.89	
	Total	103	67.87	16.57	64.64	71.11	0.341
Difficulty to work (VAS)	Control	33	6.55	1.99	5.84	7.25	
	Physiotherapy	36	6.86	2.6	5.98	7.74	
	Acupuncture	34	6.68	2.31	5.87	7.48	
	Total	103	6.7	2.3	6.25	7.15	0.851
Pain (VAS)	Control	33	7.15	2.06	6.42	7.88	
	Physiotherapy	36	7.19	2.1	6.49	7.9	
	Acupuncture	34	7.12	2.04	6.41	7.83	
	Total	103	7.16	2.05	6.76	7.56	0.988
Stiffness (VAS)	Control	33	6.97	2.72	6	7.93	
	Physiotherapy	36	7.06	2.76	6.12	7.99	
	Acupuncture	34	6.91	2.8	5.93	7.89	
	Total	103	6.98	2.73	6.45	7.51	0.976
Depression (VAS)	Control	33	6.7	2.74	5.72	7.67	
	Physiotherapy	36	7.53	2.78	6.59	8.47	
	Acupuncture	34	7.09	2.77	6.12	8.05	
	Total	103	7.12	2.76	6.58	7.66	0.461

Note: Sig.: Significance; S-FIQ: Spanish Fibromyalgia Impact Questionnaire; VAS: Visual Analogue Scale from 0 to 10.

**Table 2:** Outcome scores for each group at baseline (week 0), post-intervention (week 6) and at follow-up (week 13): mean and standard deviation.

Outcome Measure	Group	*N*	Baseline (Mean ± SD)	Week 6 (Mean ± SD)	Week 13 (Mean ± SD)
S-FIQ (0–100)	C	33	66.42 ± 15.03	67.45 ± 17.07	69.00 ± 15.08
	PT	36	70.00 ± 17.46	62.89 ± 16.91	63.50 ± 18.44
	ACP	34	68.97 ± 16.96	62.50 ± 18.09	63.62 ± 16.29
Difficulty to work (VAS)	C	33	6.55 ± 1.99	6.79 ± 2.47	7.03 ± 2.47
	PT	36	6.86 ± 2.60	6.31 ± 2.23	6.56 ± 2.41
	ACP	34	6.68 ± 2.31	6.26 ± 2.33	6.85 ± 2.51
Pain (VAS)	C	33	7.15 ± 2.06	7.18 ± 2.37	7.18 ± 2.14
	PT	36	7.19 ± 2.10	6.69 ± 2.40	6.64 ± 2.37
	ACP	34	7.12 ± 2.04	6.53 ± 2.00	6.62 ± 2.15
Stiffness (VAS)	C	33	6.97 ± 2.72	6.97 ± 2.64	7.30 ± 2.28
	PT	36	7.06 ± 2.76	6.31 ± 2.86	6.22 ± 2.93
	ACP	34	6.91 ± 2.80	6.00 ± 2.96	6.50 ± 2.67
Depression (VAS)	C	33	6.70 ± 2.74	6.52 ± 3.03	6.67 ± 2.84
	PT	36	7.53 ± 2.78	6.08 ± 2.99	6.31 ± 3.18
	ACP	34	7.09 ± 2.77	6.29 ± 3.15	6.15 ± 3.17

Note: S-FIQ = Spanish Fibromyalgia Impact Questionnaire; VAS: Visual Analogue Scale from 0 to 10; C: Control; PT: Physiotherapy; ACP: Acupuncture.

**Table 3:** Between-group differences in weeks 0, 6 and 13 (derived from one-way ANOVA in each week).

Outcome Measure	Week	F-Test	*p*-Value
S-FIQ (0–100)	0	1.09	0.34
	6	0.85	0.43
	13	1.19	0.31
Difficulty to work (VAS)	0	0.16	0.85
	6	0.52	0.60
	13	0.33	0.72
Pain (VAS)	0	0.01	0.99
	6	0.75	0.48
	13	0.70	0.50
Stiffness (VAS)	0	0.02	0.98
	6	1.03	0.36
	13	1.53	0.22
Depression (VAS)	0	0.78	0.46
	6	0.17	0.84
	13	0.25	0.78

Note: S-FIQ: Spanish Fibromyalgia Impact Questionnaire; VAS: Visual Analogue Scale from 0 to 10.

**Table 4:** Changes between weeks 0 and 6, 6 and 13 and 0 and13 for each group: 95% CI for the mean difference and *p*-value (derived from the mixed-model analysis).

Outcome	Group	*N*	0–6 Weeks: 95% CI for Mean Difference/*p*-Value		6–13 Weeks: 95% CI for Mean Difference/*p*-Value		0–13 Weeks: 95% CI for Mean Difference/*p*-Value	
S-FIQ (0–100)	C	33	−3.03 ± 7.91	0.45	−1.55 ± 7.93	0.70	−4.58 ± 7.41	0.22
	PT	36	7.11 ± 8.08	0.08	−0.61 ± 8.32	0.88	6.50 ± 8.44	0.13
	ACP	34	6.47 ± 8.49	0.13	−1.12 ± 8.34	0.79	5.35 ± 8.06	0.90
Difficulty to work (VAS)	C	33	−0.24 ± 1.10	0.66	−0.24 ± 1.21	0.69	−0.49 ± 1.10	0.38
	PT	36	0.56 ± 1.14	0.33	−0.25 ± 1.09	0.65	0.31 ± 1.18	0.61
	ACP	34	0.41 ± 1.12	0.47	−0.59 ± 1.17	0.32	−0.18 ± 1.17	0.76
Pain (VAS)	C	33	−0.03 ± 1.09	0.96	0.00 ± 1.11	0.99	−0.03 ± 1.03	0.95
	PT	36	0.50 ± 1.06	0.35	0.06 ± 1.12	0.92	0.56 ± 1.05	0.29
	ACP	34	0.59 ± 0.98	0.24	−0.09 ± 1.01	0.86	0.50 ± 1.01	0.33
Stiffness (VAS)	C	33	0.00 ± 1.32	0.99	−0.33 ± 1.21	0.59	−0.33 ± 1.24	0.59
	PT	36	0.75 ± 1.32	0.26	0.08 ± 1.36	0.90	0.83 ± 1.34	0.22
	ACP	34	0.91 ± 1.40	0.20	−0.50 ± 1.37	0.47	0.41 ± 1.32	0.54
Depression (VAS)	C	33	0.18 ± 1.42	0.80	−0.15 ± 1.44	0.84	0.03 ± 1.37	0.97
	PT	36	1.44 ± 1.44	0.04	−0.22 ± 1.45	0.76	1.22 ± 1.40	0.09
	ACP	34	0.79 ± 1.44	0.27	0.15 ± 1.53	0.85	0.94 ± 1.44	0.20

Note: S-FIQ = Spanish Fibromyalgia Impact Questionnaire; VAS: Visual Analogue Scale from 0 to 10; C: Control; PT: Physiotherapy; ACP: Acupuncture.

## Data Availability

The data underlying this article cannot be shared publicly to maintain the privacy of individuals that participated in the study. The data will be shared on reasonable request to the corresponding author.

## Supplementary Materials

The following are available online at https://www.mdpi.com/article/10.3390/jcm10173765/s1, Table S1: Descriptions of the interventions conducted following the Template for Intervention Description and Replication (TIDieR).
