# Supplementary material for: Effects of Physiotherapy vs. Acupuncture in Quality of Life, Pain, Stiffness, Difficulty to Work and Depression of Women with Fibromyalgia: A Randomized Controlled Trial
Source: J Clin Med. 2021 Aug 24;10(17):3765. doi: 10.3390/jcm10173765 (PMC8432086; doi:10.3390/jcm10173765)
Supplement: Supplementary file 1 [file jcm-10-03765-s001.zip › jcm-1307884-supplementary.pdf]

Supplemental Table S1. Descriptions of the interventions conducted following the Template for Intervention Description and Replication (TIDieR)

Item nº	Item<br>Physiotherapy group
	**BRIEF NAME**
**1.**	Physiotherapy intervention: core stability exercise programme
	**WHY**
**2.**	The exercise programme aimed to work on core stability. This type of exercise aims to improve proximal stability to allow better upper and lower limb movement (1). Proximal stability is essential for correct body function. If the stabilisation system does not function and there is no balance between stabilising and mobilising muscles, muscle imbalance and movement dysfunction will occur. This will lead to mechanical stress on bone structures and the neuromuscular system and have a negative impact on pain, joint stiffness and general well-being among others (2).<br><br>Fibromyalgia symptoms include muscle asymmetry and problems with antalgic posture (3) and a core stability programme can provide a complete and globally beneficial exercise programme (4) for women with this condition.
	**WHAT**
**3.**	**Materials:** Exercise mats, pillows.
**4.**	**Procedures:** Before the beginning of the treatment sessions, the basic principles of core stability exercises were explained to the participants. These involved transverse abdominus activation, correct alignment, breathing and correct positioning of the pelvis in neutral. The exercise programme included 7 exercises performed in crook lying and side lying position on exercise mats. Cushions were used to allow the participants to be comfortable in all positions. The exercises in the crook lying position were core activation with breathing, single leg lift with knees bent, single leg slides, bridging and knee drop sideways. The exercises completed in side lying included hip external rotation with knees bent and hip abduction with knees straight. After each session gentle stretching of the lower limbs and lumbar spine were performed.<br><br>In all sessions, the physiotherapist guided the participants and reinforced the importance of the coordination of breathing and core activation. At all times during the intervention the no pain rule was respected and fatigue was avoided.
	**WHO PROVIDED**
**5.**	A qualified physiotherapist provided the treatment. She was a member of the Spanish Chartered Society of Physiotherapists. She was trained in Kinetic Control® dynamic balance and sensorimotor system rehabilitation and the Pilates method. She also had an extensive experience in core stability training applied in rehabilitation.

	**HOW**
**6.**	All the sessions were provided face-to-face in groups of no more than 8 people
	**WHERE**
**7.**	The location were the treatment sessions were carried out were the Physiotherapy laboratory classrooms of the University of Extremadura, Badajoz, Spain.
	**WHEN and HOW MUCH**
**8.**	The physiotherapy sessions lasted for 30 minutes and were done twice a week. The treatment was conducted during 5 weeks.
	**TAILORING**
**9.**	Not applicable. The exercise programme was designed to be performed by adults independently mobile with or without mobility devices and considering the symptoms of Fibromyalgia.
	**MODIFICATIONS**
**10.**	Not applicable
	**HOW WELL**
**11.**	Intervention adherence and attendance at treatment sessions was recorded by the professional guiding the treatment.

**Item nº**	**Item**
	**Acupuncture group**
	**BRIEF NAME**
**1.**	Acupuncture treatment
**2**	**WHY**
	The Traditional Chinese Medicine interpretation of Fibromyalgia suggest that this condition is caused by an alteration of the body's energy (*qi*) and blood (*xue*) movement that leads to their deficiencies and stasis mainly in the lower levels of the body (5,6). This causes weakness, dizziness, balance impairments leading to other symptoms such as pain and decrease general wellbeing. As the Traditional Chinese Medicine aphorism says: 'when the lower is insufficient, the high stumbles'. Therefore, the treatment approach must focus on the restoration of proper flow of these elements (5,6).

Point selection was based on their therapeutic effect and the Traditional Chinese Medicine interpretation of Fibromyalgia. The selected points were: GV20 (*Baihui* 百会) which is considered as a connection of all the energy channels. GV20 is indicated to increase *qi* levels and therefore to increase energy levels; ST36 (*Zusanli* 足 三 里) which harmonises the energy and blood and has a *qi* toning and body strengthening effect. Therefore, it is indicated for the treatment of disorders that present with fatigue and weakness. Its Chinese name refers to its ability to restore strength of the body and lower limbs allowing the person to walk for a very long distance; BL60 (*Kunlun* 昆仑) which facilitates the flow of the Traditional Chinese Medicine channels. It also regulates and tones the musculoskeletal system, especially the lumbar spine and the lower limbs (7,8). All the effects attributed to these acupuncture points can contribute to improve the symptoms and well-being of the patients with Fibromyalgia and consequently their quality of life.

WHAT

3. **Materials:** Plinths, pillows, wedge cushions, cotton pads, 70% ethyl alcohol, one-time-use disposable sterile stainless steel needles (0,26x50mm) of 0.5 , 1 and 3 cun of length.

4. **Procedures**: Acupunture treatment was provided to the participants. The acupuncture points used were: GV20 (*Baihui* 百会) which is located at the highest point in the head, in the midpoint of the line connecting the apexes of the two auricles, ST36 (*Zusanli* 足 三 里) which is found on the anterior aspect of the lower leg, 3 cun (width of a person's thumb at the level of the knuckle) below the inferior edge of the patella and 1 cun from the anterior crest of the tibia and BL60 (*Kunlun* 昆仑) located on the foot, behind the external malleolus, in the depression between the tip of the external malleolus and the calcaneus tendon (7,8).

The acupuncturist explained to the participants the possible discomfort that they may experience when the needle was introduced. Besides, she described the *deQi* sensation and requested the participants to indicate when they felt it. They were informed about the secondary effects at the moment of signing the informed consent.

Patients laid supine on a treatment table with their legs exposed. The skin on the acupuncture points was prepared with 70% ethyl alcohol. All participants received 5 needle insertions in each session. One needle was inserted in GV20 point while ST36 and BL60 insertions were done bilaterally. One-time-use disposable sterile stainless steel needles (0,26x50mm) were inserted into acupuncture points. The needles used for GV20 were 0.5 cun long, 1 cun for BL60 and 3 cun long for ST36. The depth of insertion was based on the recommendations for each point (normally between 8 to 30 mm) (7) and was also marked by reaching the *deQi* sensation by the participant. The *deQi* sensation is defined as the patient feeling an ache or heaviness in the area around the needle as well as the muscle twitch response that the acupuncturist can feel during the insertion of the needle. In order to obtain the *deQi*, the acupuncture needles were also manually manipulated with bi-directional rotational movements. The needles

	remained in place for 20 minutes and there were no further manipulations during the retention time.
	**WHO PROVIDED**
**5.**	The Acupunture treatment was provided by a qualified Traditional Chinese Medicine trained acupuncturist (ACMAS Superior School of Acupuncture of Seville and Beijing University) member of the Chartered Society of Physicians of Badajoz (Spain). She also had 22 years of experience and was professor in the University of Extremadura Acupuncture and Moxibustion Postgraduate Course.
	**HOW**
**6.**	All the sessions were provided face-to-face in individual sessions.
	**WHERE**
**7.**	The location were the treatment sessions were carried out were the Physiotherapy laboratory classrooms of the University.
	**WHEN and HOW MUCH**
**8.**	The Acupunture sessions lasted for 30 minutes (needle retention period was 20 minutes) and were done twice a week. The treatment was conducted during 5 weeks.
	**TAILORING**
**9.**	Not applicable
	**MODIFICATIONS**
**10.**	Not applicable
	**HOW WELL**
**11.**	Intervention adherence and attendance at treatment sessions was recorded by the professional guiding the treatment.

REFERENCES

1. Kibler WB, Press J, Sciascia A. The role of core stability in athletic function. Sports Med. 2006;36(3):189-98.

2. Akuthota, V, Ferreiro A, Moore T, Fredericson M. Core Stability Exercise Principles. Curr Sports Med Rep. 2008. 7(1):39-44.

3. Mitani Y,Fukunaga M, Kanbara K, Takebayashi N, Ishino S, Nakai Y. Evaluation of psychophysiological asymmetry in patients with fibromyalgia syndrome. Appl Psychophysiol Biofeedback. 2006 Sep;31(3):217-25.

4. Bliss L, Teeple P. Core stability: the centerpiece of any training program. Curr Sports Med Rep. 2005;4(3):179-83.

5. Zheng L, Faber K. Review of the Chinese medical approach to the management of fibromyalgia. Curr Pain Headache Rep. 2005 Oct;9(5):307–12.

6. Cao H, Liu J, Lewith GT. Traditional Chinese Medicine for treatment of fibromyalgia: a systematic review of randomized controlled trials. J Altern Complement Med [Internet]. 2010;16(4):397–409. Available from: http://www.pubmedcentral.nih.gov/articlerender.fcgi?artid=3110829&tool=pmcentrez&rendertype=abstract

7. Cobos R, Vas J. [Manual de Acupuntura y Moxibustión] [Spanish]. 1st ed. Beijing: Ediciones Morning Glory Publishing; 2000.

8. Hempen C-H. Altas de Acupuntura. 1st editio. Barcelona: Editorial Paidotribo; 2014.
